# Serotonin Receptor *HTR3A* Gene Polymorphisms rs1985242 and rs1062613, E-Cigarette Use and Personality

**DOI:** 10.3390/ijerph19084746

**Published:** 2022-04-14

**Authors:** Aleksandra Suchanecka, Jolanta Chmielowiec, Krzysztof Chmielowiec, Grzegorz Trybek, Aleksandra Jaroń, Wojciech Czarny, Paweł Król, Jolanta Masiak, Anna Grzywacz

**Affiliations:** 1 Independent Laboratory of Health Promotion, Pomeranian Medical University in Szczecin, 11 Chlapowskiego St., 70-204 Szczecin, Poland; o.suchanecka@gmail.com; 2 Department of Hygiene and Epidemiology, Collegium Medicum, University of Zielona Góra, Zyty 28 St., 65-046 Zielona Gora, Poland; chmiele1@o2.pl (J.C.); chmiele@vp.pl (K.C.); 3 Department of Oral Surgery, Pomeranian Medical University in Szczecin, 72 Powstanców Wlkp. St., 70-111 Szczecin, Poland; g.trybek@gmail.com (G.T.); jaronola@gmail.com (A.J.); 4 Faculty of Physical Education, University of Rzeszów, Towarnickiego 3 St., 35-959 Rzeszów, Poland; wojciechczarny@wp.pl; 5 College of Medical Sciences, Institute of Physical Culture Studies, University of Rzeszow, St. Towarnickiego 3, 35-955 Rzeszów, Poland; pkrol@ur.edu.pl; 6 Neurophysiological Independent Unit, Department of Psychiatry, Medical University of Lublin, 20-093 Lublin, Poland; jolantamasiak@wp.pl

**Keywords:** nicotine addiction, serotonin receptor, personality traits, genetics

## Abstract

We nowadays record growing numbers of e-cigarette users. The development of nicotine dependence is a result of many factors, including genetics and personality. In this study we analyzed two polymorphisms—rs1985242 and rs1062613—in the serotonin receptor *HTR3A* gene in a group of e-cigarette users (*n* = 135) and controls (*n* = 106). Personality traits were measured using the NEO Five-Factor Inventory. The comparison of e-cigarette users with the control group indicates that the former showed significantly higher scores on the neuroticism scale and lower scores on the scales of extraversion and conscientiousness of the NEO-FFI. Homozygote variants of rs1985242 were more frequent in the study group. The results of the 2 × 3 factorial ANOVA for e-cigarette users and the control group as well as interaction between the *HTR3A* rs1985242 variants were found for the NEO-FFI conscientiousness scale. These results allow us to conclude that the combination of psychological factors and genetic data creates a possibility for making more complete models of substance use disorders.

## 1. Introduction

We nowadays record growing numbers of e-cigarette users [1], but cigarette smoking is still the most common form of tobacco use and is one of the leading causes of premature death and disability [2]. Although attempts are made to control tobacco abuse, its negative impact on society is still very high. The World Health Organization estimates that currently there are about 1 billion smokers aged 15 years and above worldwide [3]. An estimated 24 million children aged 13–15 around the world smoke, and 13 million use smokeless tobacco [4]. Tobacco is one of the world’s greatest preventable causes of premature death, accounting for more than 8 million deaths each year [3]. The harmful effect of tobacco applies to non-smokers living with smokers, who are exposed to secondhand smoke–they are at greater risk for premature death as well [5]. Both methods of nicotine delivery—cigarette smoking and e-cigarette use—contribute to significant health and financial losses. Although many cigarette smokers want to stop smoking [6], few are successful [7,8]. Consequently, developing effective therapies that can help improve treatment outcomes, as well as methods that can prevent people from starting to smoke, remain an ongoing public health challenge. Therefore, it is important to compare people who use nicotine in various forms with people who have never became addicted.

Nicotine is a major psychoactive component of tobacco. It produces a variety of neurophysiological, motivational, and behavioral effects through interactions with nicotinic acetylcholine receptors (nAChRs) in the central nervous system. Twin, family, and adoptive studies suggest that nicotine addiction is associated with genetic and environmental factors, and the genetic factor plays an important role in the development of addiction [9,10]. Addiction develops as a result of repeated exposure to a psychoactive agent that interferes with and modifies the brain function as well as some behaviors. These effects are determined by the psychoactive agent, frequency of use, individual metabolism, and sensitivity to the agent, comorbidities, as well as environmental and epigenetic factors. Compulsive behaviors and adaptive social interactions, which are often antisocial, change in order that the substances of abuse may be acquired and ingested [11].

The neurotransmitter, 5-Hydroxytryptamine (5-HT, serotonin), plays an important role for numerous body functions. It is primarily involved in gastrointestinal motility, vasoconstriction, and regulation of brain processes, such as motivation, learning, and memory. The 5-HT receptor family contains at least seven well-characterized subtypes, namely receptors 5-HT1 to 5-HT7, and further differentiation within each subtype. Amongst the 5-HT receptor family, the 5-HT3 receptor (5-HTR3) is a unique subtype because it is a ligand-gated ion channel, whereas the others are G-protein-coupled receptors. The 5-HTR3 mediates the transport of cations, mainly sodium, potassium, and calcium [12]. In humans, five different subunits of 5-HTR3, which may form a homopentamer or heteropentamer, have been identified—A, B, C, D, and E. Each of them is associated with different pharmacological properties and clinical effects. The 5-HT3 receptor antagonists are used to treat irritable bowel syndrome, nausea, and vomiting in patients undergoing chemotherapy. Recent studies are also looking at the therapeutic potential of the abovementioned antagonists in other diseases, such as pain and inflammation, psychiatric disorders, neurodegenerative diseases, and substance abuse. The *5-HTR3* has been found to be expressed in immune cells [13]. Interestingly, neural tissue inflammation has been implicated in the modulation of addiction, and studies indicate that 5-HTR3 antagonists are involved in this process [14]. Administration of 5-HTR3 antagonists has been shown to affect reward effects and other behaviors induced by addictive substances, such as alcohol, where ethanol has been shown to interact directly with 5-HT3 receptors [15,16]. They also affect opioid withdrawal symptoms [17,18]. The 5-HT3 receptor is involved in the modulation of dopamine—the main neurotransmitter that regulates addiction [15,19]. Moreover, this modulation is also associated with depressive effects [20]. In relation to substance abuse, depression is generally known as one of the common withdrawal symptoms that occur after the cessation of substance use, which often becomes a reason for the addicted person to start using the substance again.

Genetic studies indicate an association of genes encoding the 5-HT3 receptor with substance dependence, including nicotine [21,22,23,24]. A strong interaction between genes encoding 5-HT3A and 5-HT3B subunits and the serotonin transporter gene with nicotine addiction has been reported [23]. The above genes also show an association in patients with comorbidities or addiction to more than one substance [24]. Specific polymorphic sites, namely rs1062613 and rs1985242, have been chosen for the analysis due to their location and previously reported association with nicotine addiction [24]. Polymorphism rs1062613 is located in the 5′-UTR region of the *HTR3A* gene, and its variants are associated with altered expression of encoded protein [25]. The rs1985242 is an intronic variant associated with nicotine addiction [23] and pain [26]. The A and B subunits of the 5-HT3 receptor are expressed in the limbic area associated with the neurobiology of nicotine addiction [27,28], and nicotine affects the 5-HT3 receptor activity. Although the 5-HT3 receptor and nicotinic acetylcholine receptor (nAChR) belong to the same family of ligand-gated ion channels, nicotine acts as an antagonist on 5-HTR3 and at the same time is an agonist on the nAChR receptor [29,30,31].

The neurobiology of nicotine addiction is complex, but the general theory is that it occurs mainly through nicotine-induced activation of nAChR receptors—more specifically by α4β2. Upon nAChR activation, the receptor opens and allows calcium influx, which triggers the opening of a voltage-dependent calcium channel (VDCC), which causes further calcium influx and calcium release from intracellular stores, such as the endoplasmic reticulum (ER) or mitochondria [32]. Calcium signaling is responsible for the release of numerous neurotransmitters, such as dopamine, serotonin, GABA, and glutamate, while nicotinic antagonists decrease that release [33]. The 5-HTR3 and nAChR receptors are approximately 30% homologous. Since both receptors are from the same superfamily, some physiological functions are thought to be modulated by both—for example modulation of blood pressure or pain responses [34]. The striatal nerve end has been shown to contain both 5-HTR3 and nAChR receptors, which suggests that a convergence of serotonergic and cholinergic systems takes place [35,36]. When two different receptors are located in the same nerve end, it is highly likely that those receptors will interact with each other, which is known as cross-regulation. Cross-regulation has been shown to occur between 5-HTR3 and nAChR in a calcium-dependent manner [35,37]. Activation of either 5-HTR3 or nAChR is followed by calcium influx through the receptor. It has been shown that there is a reduction in calcium response when nicotine was administered to the striatal nerve endings after a prolonged stimulation by the 5-HTR3 agonist, similar to that when the 5-HTR3 agonist was applied to the striatal nerve endings after prolonged nicotine stimulation [35]. It is believed that 5-HTR3 and nicotine interact through specific mechanisms that are yet to be fully comprehended.

Personality affects the person’s behavior and functioning through their lifetime. The Big Five [38,39,40] is a model consisting of five personality traits: openness, conscientiousness, extraversion, agreeableness and neuroticism. Differences between people are determined by these traits in relation to behavior, emotion, motivation, and cognition [41]. Neuroticism is associated with a higher propensity to mood changes, anxiety, depressive moods, and feeling of loneliness [42,43]. Openness is associated with intelligence and non-typical thinking [44]. Conscientiousness is a tendency to control impulses [45]. Extraversion is associated with excitability and sociability [46]. Agreeableness is associated with pro-social behaviors [47]. Differences in levels of above-mentioned personality traits are often analyzed in addiction studies as one of the factors influencing substance dependence [48,49,50].

The aim of this study was to analyze factors influencing nicotine dependence. We analyzed two polymorphisms in the *HTR3A* gene rs1985242 and rs1062613, and personality traits measured by means of the NEO Five-Factor Personality Inventory in a group of e-cigarette users and controls. The lack of a subsample of regular (non-e-cigarette) smokers prohibits the claim of specificity of results for e-smokers.

## 2. Materials and Methods

### 2.1. Participants

The study was conducted in the Independent Laboratory of Health Promotion, Pomeranian Medical University in Szczecin. The study group included 135 volunteers using e-cigarettes (females: 49.63%, males: 50.37%) aged 26 ± 9.44 who were somatically healthy. A history of psychosis and addiction to a substance other than nicotine were excluded. The control group comprised 106 unrelated (females: 24.53%, males: 75.47%), healthy (non-dependent and non-psychosis) Polish volunteers aged 23.79 ± 8.72 who smoked less than 30 cigarettes in their lifetime. To reduce the possibility of racial gene skewing and to overcome any potential problems due to population stratification, all subjects and controls were Caucasian.

The study was conducted in accordance with the Declaration of Helsinki principles and approved by the Ethics Committee. All subjects signed their informed consent for participating in the research. No financial or any other compensation for being part of the study was granted. The assessment process took place in a single session which lasted about 100 min. Psychologists and psychiatrists collected data for the semi structured interview. Additionally, the interviewed group was given an option to be helped by an assistant who was present in the room and who also checked if the tests were completed. All the procedures for allowing comfort and concentration were accomplished. Psychology specialists accomplished the interpretation of the tests.

### 2.2. Genetic Tests

DNA was obtained from venous blood. The genomic DNA was purified using standard methods. Samples from each subject were genotyped using the real-time PCR and fluorescence resonance energy transfer LightSNiP oligonucleotide probes on Light-Cycler 480 II (Roche Diagnostic, Basel, Switzerland) according to the manufacturer’s protocols. The fluorescence signal was plotted against temperature. Peaks were obtained at 60.3 °C for the T allele and at 64.19 °C for the A allele of rs1985242 and at 56.3 °C for the C allele and at 64.06 °C for the T allele of rs1062613.

### 2.3. Psychological Tests

The NEO Personality Inventory scale (NEO Five-Factor Inventory, NEO-FFI) is based on six dimensions for each of the five traits: extraversion (positive emotion, warmth, gregariousness, activity, excitement seeking, and assertiveness); agreeableness (tendermindedness, trust, altruism, straightforwardness, compliance, and modesty); openness to experience (fantasy, feelings, aesthetics, actions, values, and ideas); conscientiousness (deliberation, competence, dutifulness, order, achievement striving, and self-discipline); and neuroticism (anxiety, vulnerability to stress, hostility, self-consciousness, impulsiveness, and depression) [38].

### 2.4. Statistical Analysis

The concordance of the genotype frequencies and the Hardy–Weinberg equilibrium was tested using HWE software (https://wpcalc.com/en/equilibriumhardy-weinberg, accessed 3 June 2021). Associations among *HTR3A* rs1985242 and *HTR3A* rs1062613, e-cigarette users, controls, and the NEO Five Factor Inventory were analyzed using multivariate ANOVA (control and e-cigarette users × genetic trait × NEO-FFI × (control and e-cigarette users × genetic trait)) factor effects analysis. The homogeneity of variance condition was met (Levene’s test *p* > 0.05). Normality of distribution was not met for the analyzed variables. Analysis and comparison of the five-factor NEO Inventory scales was performed using the Mann–Whitney U test.

The chi square test and Cochran–Armitage trend test were used for association analysis of *HTR3A* rs1985242 and *HTR3A* rs1062613 and e-cigarette use. For these variables, the Bonferroni multiple comparisons correction was applied, and the accepted level of significance was 0.01 (0.05/5) and 0.0083 (0.05/6), respectively. All calculations were performed using STA-TISTICA 13 (Tibco Software Inc., Palo Alto, CA, USA) for Windows (Microsoft Corporation, Redmond, WA, USA) and CATT package in R for the calculation of Cochran–Armitage trend test.

## 3. Results

The frequency distributions did not accord with the HWE in the study group for both polymorphisms (Table 1).

Association analysis of genotypes and alleles of *HTR3A* rs1985242 and rs1062613 polymorphisms and e-cigarette use revealed statistically significant differences only in the Co-dominant model in frequencies of genotypes for rs1985242 (T/T 0.58 vs. 0.47; A/T 0.32 vs. 0.47; A/A 0.10 vs. 0.07; χ^2^ = 6.456; *p* = 0.0396). In contrast, there was no statistically significant difference between the control group and e-cigarette users in the Additive model (Cochran–Armitage trend test) in *HTR3A* rs1985242 (Z = −0.707, *p* = 0.480) and rs1062613 (Z = 0.044, *p* = 0.965) polymorphisms (Table 2).

In the U-Man test, there were no significant statistical differences in the control group between women (F) and men (Mn) in the components of the NEO-FFI tests (neuroticism scale F; M = 4.15 vs. Mn; M = 5.15, *p* = 0.055—not significant with Bonferroni correction; extraversion scale F; M = 7.19 vs. Mn, M= 6.89, *p* = 0.360; openness scale F; M = 5.04 vs. Mn; M = 5.03, *p* = 0.947; agreeableness scale F; M = 6.26 vs. Mn; M = 5.75, *p* = 0.231; conscientiousness scale F; M = 7.88 vs. Mn; M = 6.96, *p* = 0.053—not significant with Bonferroni correction).

The means and standard deviations of the NEO Five-Factor Inventory results in the group of e-cigarette users and controls are presented in Table 3. In comparison with the controls, the study group had significantly higher scores on the neuroticism scale (M 5.60 vs. M 4.91, *p* = 0.0117—not significant with Bonferroni correction) and lower scores on the extraversion scale (M 6.05 vs. M 6.96, *p* = 0.0014) and conscientiousness scale (M 6.05 vs. M 7.19, *p* < 0.0001).

However, Table 4 and Table 5 show the means and standard deviations of the NEO Five-Factor Inventory results in the group of e-cigarette users and controls, including the *HTR3A* rs1985242 and rs1062613 polymorphisms.

### 3.1. Conscientiousness Scale and HTR3A rs1985242

**Figure 1:**
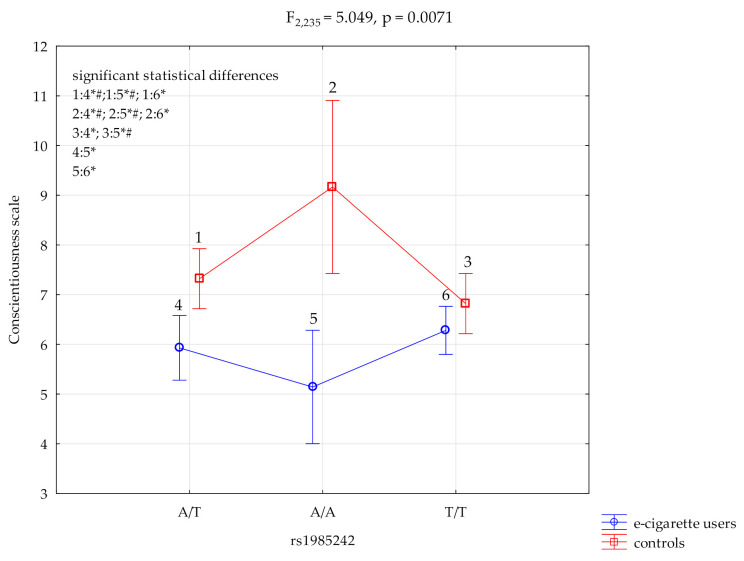
Interaction between e-cigarette users/controls, *HTR3A* rs1985242 and conscientiousness scale. * significant statistical differences, ^#^ Bonferroni correction was used, and the *p* value was reduced to 0.0083 (*p* = 0.05/6 (number of statistical tests conducted)).

The results of 2 × 3 factorial ANOVA showed a statistically significant effect of the combined factor *HTR3A* rs1985242 genotype of e-cigarette users/control (F_2,235_ = 5.05, *p* = 0.0071, η^2^ = 0.041) (Table 4, Figure 1). For the power calculation, our sample had more than an 81% power to detect the combined factor of e-cigarette users/control x *HTR3A* rs198524*2* and their interaction effect (about 4.1% of the phenotype variance). The post hoc analysis is shown in Table 4.

Post hoc LSD analysis showed a statistically significant lower score of conscientiousness scale in e-cigarette users result for the *HTR3A* rs1985242 polymorphic variant A/T M = 5.93 compared to control A/T M = 7.32, *p* = 0.0023 and A/A M = 9.17, *p* = 0.0007. The same was found for the conscientiousness scale of e-cigarette users variant A/A M = 5.14 compared to controls for A/T M = 7.32, *p* = 0.0010 and A/A M = 9.17, *p* = 0.0002 variants. Additionally, the scores were lower scores on the conscientiousness scale in e-cigarette users for T/T variant M = 6.28 versus controls A/A M = 9.17, *p* = 0.0019 (Table 6, Figure 1).

### 3.2. Conscientiousness Scale and HTR3A rs1062613

**Figure 2:**
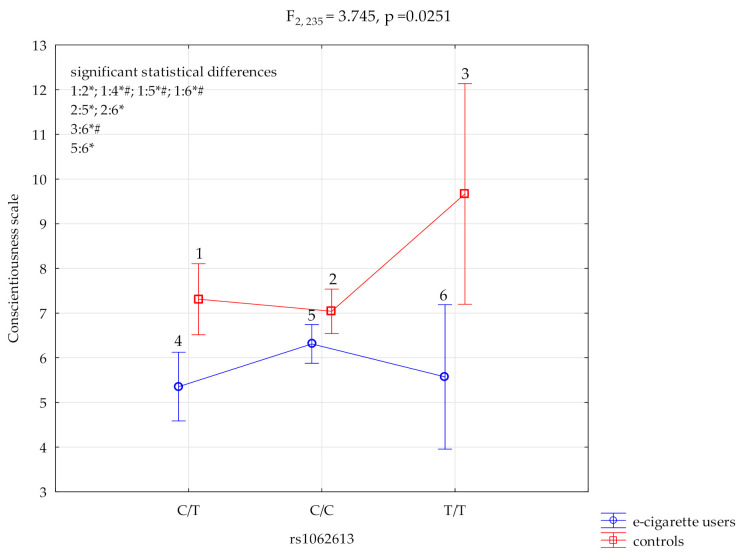
Interaction between e-cigarette users/controls, *HTR3A* rs1062613 and conscientiousness scale. * significant statistical differences, ^#^ Bonferroni correction was used, and the *p* value was reduced to 0.0083 (*p* = 0.05/6 (number of statistical tests conducted)).

The results of the 2 × 3 factorial ANOVA showed a statistically significant effect of the combined factor *HTR3A* rs1062613 genotype of e-cigarette users /control (F_2,235_ = 3.74, *p* = 0.0251, η^2^ =0.031; not significant with Bonferroni correction) (Table 5, Figure 2). For the power calculation, our sample had more than a 68% power to detect the combined factor of e-cigarette users/control x *HTR3A* rs1062613 and their interaction effect (about 3.1% of the phenotype variance). The post-hoc analysis is shown in Table 4.

In the post hoc LSD analysis, a statistically significant lower scores of conscientiousness scale in e-cigarette users were obtained for the *HTR3A* rs1062613 polymorphic variant C/T M = 5.35 compared to control C/T M = 7.31, *p* = 0.0006, C/C M = 7.04, *p* = 0.0003 and T/T M = 9.67, *p* = 0.0012. Additionally, there was a lower conscientiousness scale e-cigarette users score for T/T polymorphism M = 5.57 versus the control polymorphism T/T M = 9.67, *p* = 0.006 (Table 6, Figure 2).

## 4. Discussion

E-cigarettes, which were introduced nearly 15 years ago as a smoke-free alternative to cigarettes, are now often used as a smoking cessation aid or harm reduction method for cigarette smokers. However, they are a source of nicotine, which may lead to addiction [51,52,53,54,55,56,57]. Furthermore, the distribution of nicotine in the brain and blood of e-cigarette users is very similar to the distribution and release rate as that of cigarettes, which further promotes a smoking reward and may lead to addiction [58,59,60]. Hence, e-cigarettes serve as a non-combustive substitute for cigarettes and support nicotine dependence [61]. To study nicotine addiction in the context of substance abuse in combination with other variables is rational due to the analyzed entity being multifactorial and multigene.

Our study showed differences in the analyzed personality traits between e-cigarette users and never smokers. The study group had significantly higher levels of neuroticism and lower levels of extraversion and conscientiousness than the control group. The meta-analysis [62] of personality traits and substance use disorders found an association of alcohol use disorders with low conscientiousness, inhibition, and neuroticism; other substance use disorders were also associated with low extraversion and agreeableness. Interestingly, the strength of the association was more prominent in the study group than in the population sample, indicating the importance of proper analysis of groups with mental health disorders [63]. Similar correlations of personality traits with substance use disorders were shown in the work of Zilberman et al. [64]. To directly assess whether personality traits are a source of comorbidity, Slutske et al. [65] analyzed personality traits in a group of 18-year-olds and their correlation with nicotine, cannabis, and alcohol dependence, as well as gambling disorders assessed at the age of 21. All the analyzed disorders were associated with negative emotionality and disinhibition. Cannabis addiction was associated with low positive emotionality. The high comorbidity between the analyzed disorders was reduced when individual personality traits were assessed. Individuals with a high index of neuroticism have a higher tendency for negative affect [66], and psychoactive substance use may be a way in which this affect is discharged [67,68]. Substance use and risky, unhealthy behaviors may be a consequence of low conscientiousness and higher impulsivity [69]. This explanation does not fully clarify the relationship between personality traits and substance use disorders [62]. Some authors [66,70,71] suggest that associations of certain traits with affective states and social functioning are risk factors for substance use disorders. Gender is another important variable to take into consideration. The NEO-FFI correlates for alcohol, nicotine, and cannabis use disorders were almost the same for males and females, while gender differences were evident for gambling disorders [72,73]. All the personality traits described above and their associations with various substance use disorders are also consistent with our previous studies of e-cigarette users [48], polysubstance use disorders [49,50], and cannabis use disorders [74].

Subsequent findings concerning the genetic analysis were performed. In our study, significant differences were found in the frequency of the rs1985242 genotype, with both homozygous T/T and A/A being more frequent in the study group, and the heterozygous genotype being more frequent in the control group. Interestingly, in the controls, the differences between the heterogeneous types and the homogenous genotypes AA and TT, respectively, are more pronounced than in the group of smokers. In addition, analyzing the interaction between genotype, smoking status, and personality trait, significant differences were found for the NEO-FFI contentiousness scale, with the greatest difference between smokers and never smokers occurring for the A/A genotype of rs1985242 and for the T/T genotype of rs1062613. Both analyzed polymorphisms are located in the *HTR3A* gene, with rs1062613 being a variant that regulates translation in the open-reading frame upstream of the *HTR3A* mRNA translation initiation site [25], while rs1985242 is located in the 5’UTR region. There is not much available data concerning the genotypes analyzed in our study in the context of nicotine addiction and no data regarding e-cigarette users. However, the published literature on the subject clearly shows an association of rs1985242 and rs1062613 with nicotine addiction [23]. Both polymorphisms were part of the haplotypes associated with nicotine dependence in a sample of African Americans [AA] but not in a group of European Americans [EA] [23]. SNP rs1062613 along with the interacting variant, rs1150220, were associated with the smoking quantity in the European American sample and in the pooled sample of EA and AA participants. The authors of this study also analyzed the *HTR3B* and *5HTT* genes, concluding that interactions between the three serotonergic genes may represent interacting biological effects of nicotine on fast-acting serotonergic signaling in nicotine addiction. The results found in Chinese Han smokers further indicate the interaction of *HTR3A* and *HTR3B* genes in nicotine addiction [75]. In addition, SNP rs1062613 is associated with psychiatric disorders in individuals of European descent [76,77]. The rs1062613 C/C variant is also associated with the risk of developing mental health problems in victims of childhood sexual abuse due to low central serotonin activity [78]. When exposed to stress the C/C carriers exhibit a loss of gray matter in the hippocampus [77]. The above-mentioned SNP is of great functional importance is psychiatric diseases. In our study it was significant as a variant for the conscientiousness scale with T/T carriers having higher scores, which may be associated with higher serotonergic activity and protection against not only traumatic but difficult life situations and better emotional regulation.

Serotonin is one of the neurotransmitters that play a key role in cognitive behavioral functions, stress response, mood, appetite, and motor functions [79]. The results of our study are in line with the current literature on the subject and indicate effects of personality traits and genetic factors on nicotine addiction. However, only a few variables were analyzed jointly. Since we studied a group of patients who had been carefully assigned to particular homogeneous subgroups, at first we chose one gene for our analysis. To the authors’ best knowledge this is the first study that analyzes personality traits and two polymorphisms in the *HTR3A* gene in a group of e-cigarette users and never smokers. Nonetheless, we are aware of certain limitations of our research. Only individuals of Caucasian origin were studied, and as described before, associations of variants in the *HTR3A* gene vary between individuals of different origin.

## 5. Conclusions

The comparison of e-cigarette users with the control group of never smokers conclusively indicates that the former represented significantly higher scores on the neuroticism scale and lower scores on the scales of extraversion and conscientiousness of the NEO-FFI. Results of the 2 × 3 factorial ANOVA of e-cigarette users and the control subjects and the *HTR3A* rs1985242 variants interaction were found for the NEO-FFI conscientiousness scale. These results allow us to conclude that the combination of psychological factors and genetic data creates a possibility for making more complete models of substance use disorders.

## Figures and Tables

**Table 1:** Hardy–Weinberg equilibrium of the *HTR3A* rs1985242 and rs1062613 in the group of e-cigarette users and controls.

	E-Cigarette Users *n* = 135 Observed (Expected)	χ^2^ (*p* Value) Allele Frequencies	Controls *n* = 106 Observed (Expected)	χ^2^ (*p* Value) Allele Frequencies
*HTR3A* rs1985242				
T/T	78 (73.3)	4.291 (0.038)	50 (53.1)	2.071 (0.151)
A/T	43 (52.3)	*p* allele freq (T) = 0.74	50 (43.9)	*p* allele freq (T) = 0.71
A/A	14 (9.3)	*q* allele freq (A) = 0.26	6 (9.1)	*q* allele freq (A) = 0.29
*HTR3A* rs1062613				
C/C	97 (93.8)	4.056 (0.044)	74 (73.9)	0.006 (0.938)
C/T	31 (37.5)	*p* allele freq (G) = 0.83	29 (29.2)	*p* allele freq (G) = 0.83
T/T	7 (3.8)	*q* allele freq (T) = 0.17	3 (2.9)	*q* allele freq (T) = 0.17

**Table 2:** Frequencies of genotypes and alleles of *HTR3A* rs1985242 and rs1062613 in the group of e-cigarette users and controls.

	E-Cigarette Users	Controls	Co-Dominant Model χ^2^ (*p* Value)	OR (95% Confidence)	Additive Model Cochran-Armitage Trend Test Z (*p* Value)
*HTR3A* rs 1985242					
	*n* = 135	*n* = 106	6.456 (0.0396) *		−0.707 (0.480)
T/T	78 (57.78%)	50 (47.17%)	0.520 (0.4722)		
A/T	43 (31.85%)	50 (47.17%)		0.55 (0.32–0.95) *	
A/A	14 (10.37%)	6 (5.66%)		1.50 (0.54–4.14) *	
T	199 (73.70%)	150 (70.75%)			
A	71 (26.30%)	62 (29.25%)			
*HTR3A* rs1062613					
	*n* = 135	*n* = 106	1.289 (0.5248)		0.044 (0.965)
C/C	97 (71.85%)	74 (69.81%)	0.001 (0.9633)		
C/T	31 (22.96%)	29 (27.36%)		0.81 (0.45–1.47)	
T/T	7 (5.19%)	3 (2.83%)		1.77 (0.44–7.10)	
C	225 (83.33%)	177 (83.49%)			
T	45 (16.67%)	35 (16.51%)			

*p*—statistical significance, χ^2^—Chi^2^ test result, *n*—number of subjects, * significant statistical differences, OR—odds ratio.

**Table 3:** Analysis of NEO Five Factor Inventory results in e-cigarette users and controls.

NEO Five Factor Inventory	E-Cigarette Users (*n* = 153) M ± SD	Control (*n* = 152) M ± SD	U Mann–Whitney Z	*p* Value
Neuroticism scale	5.60 ± 2.19	4.91 ± 2.24	2.522	0.0117 *
Extraversion scale	6.05 ± 2.13	6.96 ± 1.93	−3.190	0.0014 *^,#^
Openness scale	5.10 ± 1.92	5.03 ± 1.88	0.202	0.8399
Agreeableness scale	5.60 ± 2.34	5.88 ± 2.26	−0.909	0.3632
Conscientiousness scale	6.05 ± 2.15	7.19 ± 2.25	−4.147	0.0000 *^,#^

M—mean, SD—standard deviation, U—Mann–Whitney Z-test. * significant statistical differences. ^#^ Bonferroni correction was used, and the *p* value was reduced to 0.01 (*p* = 0.05/5 (number of statistical tests conducted)).

**Table 4:** The results of 2 × 3 factorial ANOVA for e-cigarette users and controls, NEO Five Factor Inventory and *HTR3A* rs1985242.

NEO Five Factor Inventory	Group	*HTR3A* rs1985242			ANOVA (Interaction)		
		T/T (*n* = 128) M ± SD	A/T (*n* = 93) M ± SD	A/A (*n* = 20) M ± SD	E-Cigarette Users/Control x *HTR3A* rs1985242 F (*p* Value)	ɳ^2^	Power (Alfa = 0.05)
Neuroticism scale	E-cigarette users; *n* = 135	5.43 ± 2.11	6.00 ± 2.29	5.290 ± 2.33	F_2,235_ = 0.28 (*p* = 0.7561)	0.002	0.094
	Control; *n* = 106	4.88 ± 2.50	5.00 ± 2.02	4.33 ± 1.86			
Extraversion scale	E-cigarette users; *n* = 135	5.91 ± 2.11	6.42 ± 2.25	5.71 ± 1.82	F_2,235_ = 1.24 (*p* = 0.2907)	0.010	0.269
	Control; *n* = 106	6.80 ± 2.02	7.00 ± 1.86	8.00 ± 1.55			
Openness scale	E-cigarette users; *n* = 135	5.19 ± 1.58	5.21 ± 1.58	4.21 ± 1.63	F_2,235_ = 1.52 (*p* = 0.2217)	0.013	0.321
	Control; *n* = 106	4.94 ± 2.23	5.04 ± 1.43	5.67 ± 2.25			
Agreeableness scale	E-cigarette users; *n* = 135	5.88 ± 2.37	5.07 ± 2.24	5.64 ± 2.37	F_2,235_ = 1.95 (*p* = 0.1440)	0.016	0.402
	Control; *n* = 106	5.66 ± 2.38	6.10 ± 2.09	5.83 ± 2.86			
Conscientiousness scale	E-cigarette users; *n* = 135	6.28 ± 2.12	5.93 ± 2.28	5.14 ± 1.70	F_2,235_ = 5.05 *^,#^ (*p* = 0.0071)	0.041	0.814
	Control; *n* = 106	6.82 ± 2.08	7.32 ± 2.38	9.17 ± 1.60			

M—mean, SD—standard deviation. * significant statistical differences. ^#^ Bonferroni correction was used, and the *p* value was reduced to 0.01 (*p* = 0.05/5 (number of statistical tests conducted)).

**Table 5:** The results of 2 × 3 factorial ANOVA for e-cigarette users and controls, NEO Five Factor Inventory and *HTR3A* rs1062613.

NEO Five Factor Inventory	Group	*HTR3A* rs1062613			ANOVA (Interaction)		
		C/C (*n* = 171) M ± SD	C/T (*n* = 60) M ± SD	T/T (*n* = 10) M ± SD	E-Cigarette Users/Control x *HTR3A* rs1062613 F (*p* Value)	ɳ^2^	Power (Alfa = 0.05)
Neuroticism scale	E-cigarette users; *n* = 135	5.40 ± 2.28	6.19 ± 1.97	5.71 ± 1.50	F_2,235_ = 0.64 (*p* = 0.5276)	0.005	0.157
	Control; *n* = 106	4.85 ± 2.24	5.17 ± 2.25	3.67 ± 2.08			
Extraversion scale	E-cigarette users; *n* = 135	4.86 ± 1.57	6.39 ± 2.20	6.03 ± 2.12	F_2,235_ = 2.27 (*p* = 0.1059)	0.019	0.458
	Control; *n* = 106	8.67 ± 0.57	7.24 ± 2.10	6.78 ± 1.86			
Openness scale	E-cigarette users; *n* = 135	4.14 ± 1.86	5.19 ± 1.42	5.13 ± 2.05	F_2,235_ = 2.66 (*p* = 0.0723)	0.022	0.524
	Control; *n* = 106	7.00 ± 1.73	5.13 ± 1.36	4.91 ± 2.03			
Agreeableness scale	E-cigarette users; *n* = 135	6.43 ± 2.07	4.90 ± 2.24	5.76 ± 2.36	F_2,235_ = 0.66 (*p* = 0.5183)	0.006	0.160
	Control; *n* = 106	5.67 ± 4.16	5.72 ± 2.22	5.95 ± 2.23			
Conscientiousness scale	E-cigarette users; *n* = 135	6.31 ± 2.14	5.35 ± 2.18	5.57 ± 1.40	F_2,235_ = 3.74 * (*p* = 0.0251)	0.031	0.681
	Control; *n* = 106	7.04 ± 2.21	7.31 ± 2.35	9.67 ± 0.58			

M—mean, SD—standard deviation. * significant statistical differences. ^#^ Bonferroni correction was used, and the *p* value was reduced to 0.01 (*p* = 0.05/5 (number of statistical tests conducted)).

**Table 6:** Post hoc LSD (least significant difference) test of interactions between e-cigarette users/controls, *HTR3A* rs1985242, *HTR3A* rs1062613, and the conscientiousness scale.

*HTR3A* rs1985242 and NEO FFI Conscientiousness Scale						
	{1} M = 5.93	{2} M = 5.14	{3} M = 6.28	{4} M = 7.32	{5} M = 9.17	{6} M = 6.82
E-cigarette users *HTR3A* A/T {1}		0.2388	0.3935	0.0023 *^,#^	0.0007*^,#^	0.0495 *
E-cigarette users *HTR3A* A/A {2}			0.0714	0.0010 *^,#^	0.0002*^,#^	0.0111 *
E-cigarette users HTR3A T/T {3}				0.0087 *	0.0019 *^,#^	0.1719
Control *HTR3A* A/T {4}					0.0497 *	0.2498
Control *HTR3A* A/A {5}						0.0129 *
Control *HTR3A* T/T {6}						
***HTR3A* rs1062613 and NEO FFI Conscientiousness Scale**						
	**{1}** **M = 5.35**	**{2}** **M = 6.31**	**{3}** **M = 5.57**	**{4}** **M = 7.31**	**{5}** **M = 7.04**	**{6}** **M = 9.67**
E-cigarette users *HTR3A* C/T{1}		0.0341 *	0.8117	0.0006 *^,#^	0.0003 *^,#^	0.0012 *^,#^
E-cigarette users *HTR3A* C/C {2}			0.386	0.0303	0.0301 *	0.0089 *
E-cigarette users *HTR3A* T/T {3}				0.0584	0.0883	0.0067 *^,#^
Control *HTR3A* C/T {4}					0.571	0.0748
Control *HTR3A* C/C {5}						0.0411 *
Control *HTR3A* T/T {6}						

* significant statistical differences, M—mean. ^#^ Bonferroni correction was used, and the *p* value was reduced to 0.0083 (*p* = 0.05/6 (number of statistical tests conducted)).

## Data Availability

Not applicable.
